# Exploring what synchronized physiological arousal can reveal about the social regulatory process in a collaborative argumentation activity

**DOI:** 10.3389/fpsyg.2022.1042970

**Published:** 2023-01-17

**Authors:** Xiaoran Li, Wanqing Hu, Yanyan Li, Ziqi Mao

**Affiliations:** ^1^Faculty of Education, Beijing Normal University, Beijing, China; ^2^Beijing Advanced Innovation Center for Language Resources, Beijing Language and Culture University, Beijing, China

**Keywords:** synchronized physiological arousal, social regulatory process, heart rate, analysis platform, physiological synchrony

## Abstract

Combining physiological measures with observational data (e.g., video or self-reports) to further capture and understand the temporal and cyclical process of social regulation has become a trend in the field. Synchronized physiological arousal is a particularly meaningful situation in collaboration. However, little attention has been given to synchronized physiological arousal episodes and their relationship with the social regulatory process. In addition, only a few research utilized heart rate (HR) as a physiological measure in the current collaboration literature. More research is necessary to reveal the potential of HR to expand the diversity of physiological indicators in the field. Therefore, the current study aimed to explore what synchronized physiological arousal can further reveal about the social regulatory process. To achieve this goal, this study designed a collaborative argumentation (CA) activity for undergraduates (mean age 20.3). It developed an arousal-regulation analysis platform, which could automatically detect synchronized physiological arousal in HR and align them with coding challenges and social regulation based on the timeline. In total, 14 four-member groups were recruited. After analyzing both videos and HR data, several findings were obtained. First, only one-third of episodes were synchronized physiological arousal episodes, and the situations where four members were all in arousal states were rare during CA. Second, synchronized physiological arousal was more sensitive to socio-emotional aspects of collaboration as the shared physiological arousal more frequently co-occurred with socio-emotional challenges and socio-emotional regulation, while it happened the least under motivational challenges. Third, synchronized physiological arousal has also been found to be associated with the challenges being regulated. Finally, pedagogical implications were suggested.

## 1. Introduction

While the expected collaborative learning is a prolific form of learning, it is often the case that learning teams encounter diverse cognitive, socio-emotional, and motivational challenges and function poorly (O’Donnell and Hmelo-Silver, 2013). To succeed in collaboration, there is a need for group members to jointly engage in a quantity of social regulation, which refers to a group deliberately and strategically taking control of tasks through shared and negotiated regulation of cognitive, behavioral, motivational, and emotional conditions (Hadwin et al., 2018a). Understanding how social regulation occurs is meaningful for conducting high-quality collaboration. Existing investigations have been successful in identifying the important patterns of social regulation from single-channel data, for example, videos (Järvenoja et al., 2019) and chat logs (Su et al., 2018). While considering the multidimensional and complex nature of social regulation, there is an increased focus on combining physiological measures with observational data (e.g., video or self-reports) to further capture and understand the temporal and cyclical process of social regulation (Järvelä et al., 2019a; Malmberg et al., 2019a).

Recent studies utilized physiological synchrony (PS) to explore how group members’ similar simultaneous changes in physiological signals [e.g., electrodermal activity (EDA), heart rate (HR)] are related to the social regulatory process (Järvelä et al., 2019a). For example, Dindar et al. (2020) unpacked that the groups’ PS index derived from EDA is only positively related to their self-report cognitive regulation rather than behavioral, motivation, and emotional regulation. Haataja et al. (2018) indicated that the high values of PS (derived from EDA) co-occurred with frequent monitoring behavior (coded from videos). From that, these investigations attempted to reveal the sharedness and the invisible social process reflected by physiological measures during the social regulatory process (Palumbo et al., 2017; Dindar et al., 2022). However, the prominent PS measures in these studies combined the correspondence between the signals of interacting individuals during both physiological arousal and non-arousal episodes (Dindar et al., 2022). As proposed by many researchers, physiological arousal is a particularly desirable goal in learning as it accounts for learners’ cognitive and/or affective activation and is more associated with active participation (Pijeira-Díaz et al., 2018). The PS measures that do not distinguish between physiological arousal and non-arousal episodes may conceal the more meaningful moments of collaboration (Haataja et al., 2018; Malmberg et al., 2019b). Therefore, researchers call for more attention to synchronized physiological arousal episodes and their relationship with social regulation (Malmberg et al., 2019a; Pijeira-Díaz et al., 2019; Dindar et al., 2022). In addition, only a few research utilized HR to profile PS in the current literature. More research is necessary to reveal the potential of HR to measure interpersonal physiology so as to expand the diversity of physiological indicators in the field (Järvelä et al., 2019a).

The current study attempts to fill in the gaps by utilizing HR to profile the synchronized physiological arousal and exploring how the synchronized physiological arousal is related to the social regulatory process in a collaborative argumentation (CA) activity. As for studying the social regulatory process, prior research has identified two important perspectives that can be drawn upon for this study, that is, encountering challenges and social regulation focuses (Su et al., 2018; Järvenoja et al., 2019; Li et al., 2022). Social regulation is multidimensional, including the regulation of cognitive, behavioral, motivational, and emotional conditions (Dindar et al., 2020), and encountering challenges are critical moments to provoke social regulation (Järvenoja et al., 2019). Preliminary research has indicated that PS is positively related to cognitive regulation (Dindar et al., 2020) and monitoring behavior (Haataja et al., 2018). Little is still known about the relationship between interpersonal physiology and encountering challenges. Further investigation combining physiological measures with observational data on the encountering challenges and social regulation focuses could shed light on this multifaced process. Therefore, this study uses HR to profile synchronized physiological arousal and explores the differences in synchronized physiological arousal when groups (a) face different types of challenges (e.g., cognitive, socio-emotional, and motivational challenges) and (b) engage in different social regulation focuses (e.g., metacognitive vs. socio-emotional regulation). In addition, the association between the challenge being regulated and synchronized physiological arousal is also investigated. From that, the study aims to unpack what synchronized physiological arousal can reveal about the social regulatory process. To achieve the goal, this study designed a CA activity titled “is the fast diet a healthy way to lose weight?” for undergraduates and developed an arousal-regulation analysis platform in which synchronized physiological arousal is detected through HR and is aligned with challenge and social regulation behavior coding from videos based on the timeline.

## 2. Literature review

### 2.1. Encountering challenges and social regulation focus on collaborative learning

High-quality collaborative learning is hard to achieve for the reason that learning teams could encounter diverse cognitive, socio-emotional, and motivational challenges (Näykki et al., 2014). Researchers have strived to uncover what challenges students encounter during the collaboration for better understanding and supporting this process (Järvenoja et al., 2019). For example, Koivuniemi et al. (2017) interviewed 107 first-year higher education students about their challenge experience in collaborative learning situations. They found that students encountered diverse challenges in collaboration, for example, concentration (motivational challenge), lacking prior knowledge (cognitive challenge), tiredness (wellbeing challenge), and frustration (emotion challenge). Recently, encountering challenges has become an important perspective in studying the social regulatory process because they are regarded as critical moments to provoke social regulation behavior (Järvelä et al., 2019b). Researchers focus on what social regulation is triggered by encountering challenges. Järvenoja et al. (2019) videotaped a 6-week mathematics course for 62 higher education students and explored how students activate group-level socio-emotional regulation in the face of diverse challenges. Through building a process model, they found that socio-emotional regulation only occurred after emotional and motivational or social context challenges. In a 2-month multimedia course for 103 teacher education students, Malmberg et al. (2015) collected the self-report data from a Virtual Collaborative Research Institute (VCRI) learning environment. They investigated how groups’ social regulatory processes progressed as collaboration developed. In the process model, they revealed that the strategies adopted by high-performing groups to regulate challenges shifted from cognitive regulation to emotional regulation, while the low-performing groups stagnated at cognitive regulation.

Apart from encountering challenges, the social regulation focus is another prominent perspective to examine the social regulatory process. Some investigations trace the process according to social regulation focus, such as cognitive, emotional and motivational regulation, and unpack important patterns contributing to a successful collaboration (Järvelä et al., 2019b). Su et al. (2018) analyzed the chat logs from Tencent QQ generated in a semester-long online collaborative language learning course. They conducted sequential analysis and found that the high-performing undergraduate groups demonstrated more significant sequential links between socio-emotional regulations than the low-performing groups. In Ucan and Webb’s (2015) investigation, they examined primary students’ utterances from a 7-week science course, and by comparing the frequency, they indicated that students not only engaged in metacognitive regulation but can also actively regulate emotional and motivational states to maintain effective group functioning. Overall, the two important perspectives identified in previous studies, encountering challenges and social regulation focus, provide the basis for this study to explore the social regulatory process.

### 2.2. Physiological synchrony and physiological arousal in the social regulatory process

Although, as shown in previous studies, data from one channel (e.g., video or chat logs) can provide valuable information about the social regulatory process in terms of strategic adaptation in the face of challenges and social regulation focus, the advances in technology and new data-capturing devices offer novel ways to examine and understand the role of these processes across learning contexts, age groups, and tasks (Järvelä et al., 2019a). Studies indicate that in interactive and collaborative learning, an individual learners’ physiological activity could be dependent upon group members (Dindar et al., 2020). This interpersonal physiology, which is defined as any interdependent or associated activity identified in the physiological processes of two or more individuals (Palumbo et al., 2017), can reflect invisible social processes co-occurring with observable interactions (Malmberg et al., 2019a). Therefore, researchers have explored how PS, one prominent indicator of interpersonal physiology, is related to the social regulatory process, and preliminary findings were obtained. For example, Dindar et al. (2019) investigated the relationship between monitoring behavior and PS between the collaborating group members. They found that the relationship between PS and shared monitoring might be dependent on task type. That is, a significant relationship was observed in one session, whereas no significant relationship was observed in the other session. In Sobocinski et al.’s (2020) research, they explored the group-level physiological state transitions during collaboration. They found that the group-level physiological state transitions were positively correlated with on-track sequences.

However, some researchers proposed that these PS measures that do not distinguish between physiological arousal and non-arousal episodes may hide more meaningful moments of collaboration (Malmberg et al., 2019a; Pijeira-Díaz et al., 2019; Dindar et al., 2022). In general, arousal can be described as a state of physiological wakefulness with emotional reactivity, enhanced cognitive processing, and increased motor activation (De Lecea et al., 2012; Critchley et al., 2013). Previous empirical research has proven that physiological arousal is positively related to a learner’s achievement (Pijeira-Díaz et al., 2018) and increased mental effort (Malmberg et al., 2019b). Therefore, the synchronized arousal of two or more collaborating members, referring to synchronized physiological arousal, is a particularly meaningful situation and is informative of collaboration (Malmberg et al., 2019a; Pijeira-Díaz et al., 2019; Dindar et al., 2022). The scant research that has been conducted only recently has begun to explore synchronized physiological arousal during the social regulatory process. In Malmberg et al.’s (2019a) exploratory research, they investigated how the simultaneous arousal between two or more group members revealed social regulation. Their findings were that most of the collaborative interaction during simultaneous arousal was low level, and social regulation was not observed. However, when the interaction was high level, and social regulation was present; when the interaction was confused, it included monitoring behavior. This investigation provides preliminary insight into the meaningfulness of synchronized physiological arousal in social regulation. More explorations are still needed to reveal what additional valuable information the synchronized physiological arousal can provide about the social regulatory process. Furthermore, previous research mainly utilized EDA to profile learners’ PS, and only a few research adopted HR (e.g., Sobocinski et al., 2020, 2022). More research is necessary to reveal the potential of HR to measure interpersonal physiology so as to expand the diversity of physiological indicators in the field (Järvelä et al., 2019a).

### 2.3. Using heart rate to profile synchronized physiological arousal

Although previous research has successfully utilized HR to profile PS in collaborative learning (e.g., Sobocinski et al., 2020, 2022), little study has adopted HR to profile synchronized physiological arousal in a collaborative learning context. A major approach to detecting physiological arousal is measuring responses of the autonomic nervous system (ANS), which consists of sympathetic and parasympathetic branches, and primarily serves a regulatory function by helping the body adapt to internal and environmental demands (Kreibig, 2010; Roos et al., 2021). Heart rate (HR) and electrodermal activity (EDA) are popular measures derived from the ANS (American Psychiatric Association, 2000). Several advantages of using ANS response in educational settings have been established. First, it is difficult for individuals to mask or control the ANS reactions, thereby creating the possibility of more objectively gauging learner’s arousal compared to self-reports (Pijeira-Díaz et al., 2019). In addition, the continuous data resulting from ANS response allow for temporal and dynamic analysis of emotional and cognitive processes of learning, some of which are even executed outside of learners’ awareness (Mendes, 2009).

Heart rate (HR) is a primary indicator of ANS response (Kreibig, 2010; Roos et al., 2021). Research in the psychophysiology field has confirmed that increases in HR reactivity may reflect arousal and have been used as popular measures for detecting psychological alertness in facing stimulus (Kreibig, 2010; Kreibig et al., 2012; Critchley et al., 2013). Mason et al. (2018) traced learners’ HRs when they read web pages about the health risks of mobile phone use with different reliability, for example, personal blog, online magazine, and academic journal (all including 420 words). They treated the increased HR as a state of arousal. Then they explored how learners’ arousal in the HR, while reading was correlated with post-reading comprehension scores. Likewise, in the investigation of Shalom et al. (2015), they attempted to explore differences in physiological arousal between high- and low-social anxiety participants when they engaged in different communication situations (computer-mediated vs. face-to-face communication). Participants’ HRs under the two conditions were collected. Arousal was identified when participants’ HR was higher than baseline, and Shalom et al. (2015) found that participants with different levels of social anxiety experienced different arousals in two situations. Previous studies that use HR to profile physiological arousal at the individual level hereby provide a basis for further exploring synchronized arousal in groups.

Therefore, the current research attempts to fill in the gaps by utilizing HR to profile the synchronized physiological arousal and exploring how the synchronized physiological arousal is related to the social regulatory process in terms of two important perspectives identified in previous research, encountering challenges and social regulation focus. The following four specific research questions (RQs) guide this study:

RQ1: How often do group member’s synchronized physiological arousal happen during the CA?

RQ2: What are the differences in synchronized physiological arousal episodes when groups face different types of challenges (e.g., cognitive, socio-emotional, and motivational challenges)?

RQ3: What are the differences in synchronized physiological arousal episodes when groups engage in different social regulation focuses (e.g., metacognitive vs. socio-emotional regulation)?

RQ4: How does synchronized physiological arousal relate to the challenge being regulated?

## 3. Materials and methods

### 3.1. Participants

A total of 56 (mean age 20.3 years, 51 females) undergraduates from a normal university in Beijing, China were recruited to involve in a CA activity titled “Is the fast diet a healthy way to lose weight?” To recruit the participants, the researchers posted announcements on the school’s online forum and offered a free lunch ticket for participation. Participants primarily majored in education and foreign language, and their standpoints on the fast diet (whether the fast diet is a healthy way to lose weight? Yes or no) were investigated through an online questionnaire before the activity. Then, the participants were randomly assigned into 14 four-member groups according to their standpoints, which consisted of two supporters and two opponents of the fast diet in each. All participants signed the Ethical Consent Form before the activity, representing that they were informed of the research purpose, confidentiality, and right to withdraw from the study.

### 3.2. Collaborative argumentation activity

**FIGURE 1:**
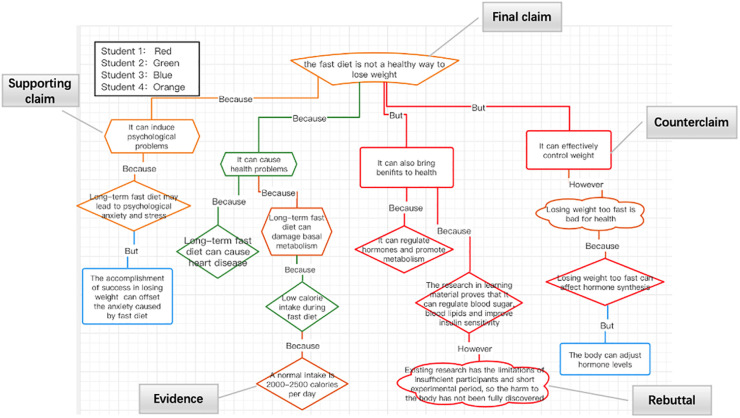
Group argument diagram.

**FIGURE 2:**
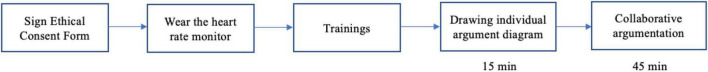
Experimental procedure.

This study was carried out in CA context. CA is a productive form of collaborative learning, which has been proven to deepen students’ understanding of complex subject concepts (Wecker and Fischer, 2014) and improve critical thinking (Ngajie et al., 2020) through a set of interactions to convince others of their arguments’ validity (Krummheuer, 1995). As the task required, students with contrary standpoints on the fast diet participated in the activity titled “Is the fast diet a healthy way to lose weight?,” aiming to reach a consensus through CA and complete a group argument diagram (Figure 1), which includes claim (final claim, supporting claim, and counterclaim), evidence, and rebuttal elements (Toulmin, 1958). This face-to-face activity was supported by an online collaborative diagramming platform.^1^ Group member used their computers to log in to the platform and synchronously edited the shared group argument diagram. Before CA, participants were trained in drawing argument diagrams and given 15 min to draw an individual argument diagram based on their existing standpoints in which students could read providing material about the nutrients and prepare for CA. Afterward, a 45-min CA was conducted in which students persuaded opponents, reached a consensus, and finished the group argument diagram (for the procedure, see Figure 2).

### 3.3. Data collection

Videos and participants’ HRs were collected in this study. The activity took place in a classroom-like research space with 360-degree cameras; therefore, the process of CA was videotaped for each group. Scosche Rhythm + armband HR monitor (Valencell, Inc., Raleigh, NC, USA) was used to record each participant’s HR continuously and unobtrusively during the whole activity. The Scosche Rhythm + is a precision biometrics apparatus to monitor HR and has a dual-mode processor in which the HR data can simultaneously transmit to multiple ANT + displays or Bluetooth-enabled devices. The former was chosen as more stable for offline processing, given our exploratory intentions. Before the activity, the Scosche Rhythm + armband HR monitor has installed on each participant’s non-dominant arm, and the sampling frequency of the sensor is 1 Hz (one sample per second). After excluding the groups with incomplete HR data, nine groups with 36 participants were finally included in this study. The total duration of valid CA was approximately 7.2 h (mean 48.6 min, SD 4.1 min).

### 3.4. Data analysis

#### 3.4.1. Video analysis

The purpose of video analysis was to identify the challenges and social regulatory behavior during CA. Videos of nine groups were analyzed by Nvivo 12 software, and the analysis unit was 30-s episodes. This time-based segmentation allowed a temporally unfolding overview of the group situations and provided a manageable and consistent unit of analysis (Järvenoja et al., 2019). There were two steps in coding the videos. The first step was to code the challenges and social regulatory behavior separately. In the second step, if a challenge is regulated by certain social regulatory behavior, an additional code as “challenge being regulated” will be given to the challenge; otherwise, a code as “challenge not being regulated” will be given.

Specifically, two coding schemes were utilized to code challenges and social regulatory behavior. For challenges, the coding scheme was adopted from Hadwin et al. (2018b), in which they identify five broad types of challenges that students encounter in various collaboration settings, namely, motivational, socio-emotional, cognitive, metacognitive, and environmental challenges. The current study integrated the metacognitive challenge into the cognitive one because it occurred less frequently (Table 1). For social regulation, the coding scheme referred to previous research by Ucan and Webb (2015), in which they have defined and conceptualized two main social regulation categories as metacognitive regulation and emotional and motivational regulation. The current study combined emotional and motivational regulation into socio-emotional regulation for the reason that they served a similar function in maintaining a productive socio-emotional climate in this investigation (Table 2). Notably, 25% of data from the CA videos were randomly chosen for inter-coding. Two researchers of this study coded the video independently, and Cohen’s kappa coefficient was calculated to judge the inter-rater reliability of the coded variables. The kappa values of challenges, social regulation, and challenges whether being regulated were 0.931, 0.864, and 0.917, respectively.

**TABLE 1:** Coding scheme for challenges.

Coding category	Description	Examples
Cognitive challenge	Cognitive challenges refer to difficulties in achieving a shared understanding of the task and domain, or in choosing effective solution paths and strategies.	I lack the knowledge to judge which arguments to follow. I highly doubt the credibility of this evidence. I think our discussion just now was meaningless because you guys didn’t get our points. We’re totally stuck, and no one can convince anyone.
Motivational challenge	Motivational challenges revolve different personal priorities, self-efficacy or different participation levels. Typically, these challenges result in declines in effort, engagement or participation.	Some students played with mobile and didn’t participate in the activity for a while. A: I still don’t understand the differences between the counterclaim and rebuttal. B: Whatever, it doesn’t matter.
Socio-emotional challenge	Socio-emotional challenges refer to challenges in creating and maintaining a positive climate, such as relational problems associated with achieving psychological safety, communicating effectively, and navigating power relationships.	I’m really speechless. We are totally not in the same mind…Oh my, I’m out of ideas. I quit. I think you are very ridiculous to require me to provide all the literature? I’m not here to teach you how to do a literature review.
Environmental challenge	Environmental challenges related to external conditions surrounding collaborative work such as technology or physical discomfort caused by environments.	I tried many times, but I don’t know how to add “because” on the edge. The room is too hot to breathe.

**TABLE 2:** Coding scheme for social regulation.

Coding category	Description	Examples
Metacognitive regulation	Group members jointly enact various behavior pertaining to planning, monitoring, task-specific strategies using and evaluation to regulate the cognitive aspects of the learning process.	How about you two write the supporting ideas and we conclude the opposing ones. How much time is left? It seems that there are only 15 min. We need to draw the diagram immediately. Hey, let’s discuss this issue from another angle. The content of our argument diagram is quite rich.
Socio-emotional regulation	Group members jointly enacted various behaviors to manage emotional states and promote motivation, alternatively, to maintain a productive socio-emotional climate.	From my body shape, you guys could tell that I have no right to talk about losing weight. Could you talk more about your opinion? I agree with your points. They are very reasonable.

#### 3.4.2. Heart rate analysis

To analyze HR data and align it with video data on the timeline, the current study developed an arousal-regulation analysis platform with two main functions, namely, identifying synchronized physiological arousal episodes in HR and aligning it with coding challenge or social regulation based on the timeline. Using the platform, two types of files needed to be prepared, separate HR files (downloaded from the HR receiver) of each member in a group and the group’s video coding file. The timeline of the two types of files should be aligned. After uploading the two types of files, the platform can automatically identify the synchronized physiological arousal episodes within a group and correspond them with the group’s coding challenge or social regulation based on the timeline. While providing visual presentations, this platform can also output result files for further statistical analysis. The realization of the key steps in the platform will be elaborated on next.

##### 3.4.2.1. Identifying synchronized physiological arousal episodes

**FIGURE 3:**
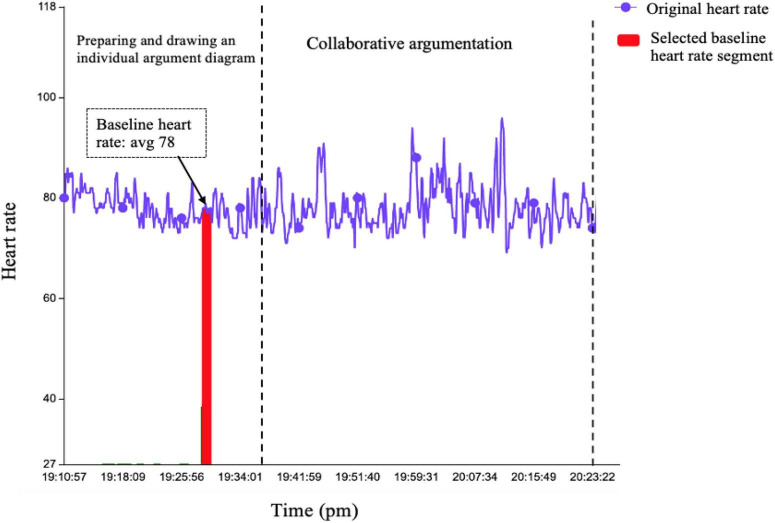
Computing individual student’s baseline heart rate (HR) [from 19:10:57 to 19:36:52 p.m., one student in group 4 was at the first stage as reading material and drawing the individual argument diagram, and from 19:36:52 to 20:23:22 p.m., she engaged in collaborative argumentation (CA). The baseline HR is 78, the average score of the selected baseline HR segment].

Before acquiring synchronized physiological arousal episodes in a group, each student’s arousal episodes needed to be identified first. Based on previous research in the psychophysiology field (Mendes, 2009; Shalom et al., 2015; Mason et al., 2018), the key point to identifying individuals’ arousal was to define baseline HR and find the episodes in which the HR was higher than baseline during the CA. Therefore, to automatically obtain an individual’s baseline HR, this study regarded the first stage of activity, that is, 15 min of reading material and drawing an individual argument diagram, as the data set for finding baseline HR because participants completed the task calmly and independently without any interference at this stage. The HR data were calculated using a moving window approach, with a window width of 1 min and a moving step of 1 s, the sampling interval of the sensor, to find the segment with the smallest standard deviation and longest duration, which represented a more stable HR and was closer to the resting HR. The average HR within the selected segment was regarded as the baseline HR. One-third of the participants were randomly chosen to verify the selected baseline HR with their actual resting HR, and the accuracy rate was 93.8% (Figure 3).

**FIGURE 4:**
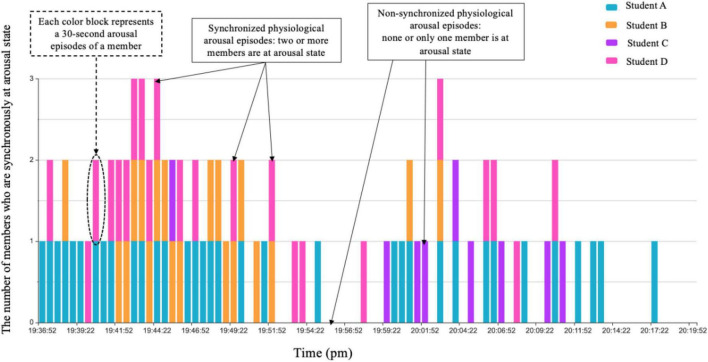
Identifying synchronized physiological arousal episodes within a group based on timeline [group 4 had four members and engaged in collaborative argumentation (CA) from 19:36:52 to 20:23:22 p.m. The *x*-axis represents time, and the *y*-axis represents the number of members who are synchronously in an arousal state. The episodes with superimposed color blocks are the synchronized physiological arousal episodes].

After determining the baseline HR, the next step was to find the individual arousal episodes during CA in which the HR was higher than the baseline. Since the study design included events that unfolded over time and there were specific time-locked events of interest, that is, coding challenges or social regulation in each 30-s episode (Mendes, 2009; Malmberg et al., 2019a), the platform also divided the individual HR data during CA into 30-s segments corresponding to the video. If the average HR within 30-s segments was higher than baseline, segments were identified as arousal episodes. Each participant’s arousal episodes were thus obtained. After aligning all members’ arousal episodes according to the timeline, the synchronized physiological arousal episodes of a group were acquired in which two or more members were synchronously in an arousal state (Figure 4).

##### 3.4.2.2. Aligning it with coding challenges or social regulation

**FIGURE 5:**
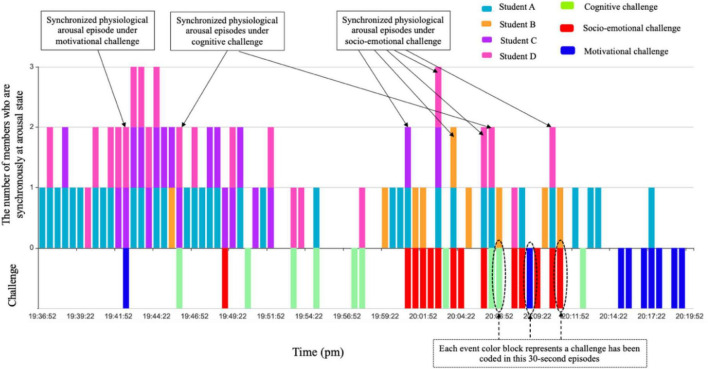
How group members’ physiological arousal synchronized under different challenges (example of group 4. The episodes where two or more arousal color blocks and event color blocks are superimposed are synchronized physiological arousal episodes under challenges).

**FIGURE 6:**
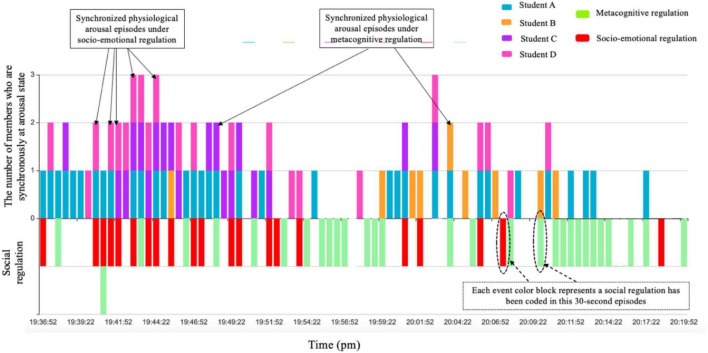
How group members’ physiological arousal synchronized under different social regulations (example of group 4. The episodes where two or more arousal color blocks and event color blocks are superimposed are synchronized physiological arousal episodes under social regulation).

Since the segmented episodes of HR were consistent with the video, the last key step was to further align the group’s arousal episodes with coding challenges or social regulation to reveal how group members’ physiological arousal in HR synchronized under different conditions in terms of facing diverse challenges (cognitive, socio-emotional, motivational, and environmental challenges) (Figure 5) as well as engaging in different social regulation focuses (metacognitive vs. socio-emotional regulation) (Figure 6).

Following previous research, this study regarded episodes in which two or more members were in the arousal state as synchronized physiological arousal episodes (Palumbo et al., 2017; Malmberg et al., 2019a). Although the visualization in the platform could provide an intuitive representation of a group, the platform also outputted the result file to answer the research question. The resulting file included information about what challenge or social regulation was coded as well as the number of members who are synchronously in an arousal state in each 30-s episode during CA. To answer RQ1, the descriptive statistics of frequency and percentage of episodes with different arousal numbers (from none to four members) during the CA were presented. For RQ2 and RQ3, the frequency and percentage of episodes with different arousal numbers under different challenges and social regulations were first given. Then, the chi-square test was utilized to analyze the differences in the frequency of synchronized physiological arousal episodes when faced with different challenges and engaged in different social regulation focuses. Similarly, the RQ4 chi-square test was utilized to examine how does synchronize physiological arousal relates to the challenge being regulated. To ensure the accuracy of the results in RQ2–RQ4, episodes containing multiple challenges or social regulation codes were excluded.

## 4. Results

### 4.1. How often does group members’ synchronized physiological arousal happen during collaborative argumentation?

Since the overall valid data were approximately 7.2 h, there were in total 867 30-s episodes calculated during CA of nine groups. Table 3 presents the frequency and percentage of episodes with different arousal numbers (from none to four members). It provides an overall picture of how group members’ physiological arousal synchronized during collaborative argumentation. As shown in Table 3, the majority of episodes (609, 70.24%) were non-synchronized physiological arousal episodes (none or 1), and only one-third of episodes (258, 29.76%) were synchronized physiological arousal episodes (two or more). Episodes with four members who were all in arousal states were extremely rare (2, 0.23%).

**TABLE 3:** Frequency and percentage of episodes with different arousal numbers (from none to four members) during collaborative argumentation (CA) activity.

	None	1	2	3	4	Total episodes
Frequency of episodes	255	354	225	31	2	867
Percentage	29.41%	40.83%	25.95%	3.58%	0.23%	100.00%

### 4.2. What are the differences in synchronized physiological arousal episodes when groups face different types of challenges (e.g., cognitive, socio-emotional, and motivational challenges)?

Table 4 shows the frequency and percentage of challenge episodes with different arousal numbers. During collaborative argumentation, there were 114 challenge episodes, with the cognitive challenge (CC, 48, 42.1%), socio-emotional challenge (SC, 42, 36.8%), and motivational challenge (MC, 22, 19.3) occurring more frequently, while environmental challenges were rare (EC, 2, 1.8%). Therefore, environmental challenges were excluded in the following analysis.

**TABLE 4:** Frequency and percentage of challenges episodes with different arousal numbers (from none to four members).

	None		1		2		3		4		Total	
	f	%	f	%	f	%	f	%	f	%	f	%
CC	15	31.3%	22	45.8%	10	20.8%	1	2.1%	0	0.0%	48	42.1%
SC	7	16.7%	16	38.1%	15	35.7%	4	9.5%	0	0.0%	42	36.8%
MC	12	54.5%	8	36.4%	2	9.1%	0	0.0%	0	0.0%	22	19.3%
EC	1	50.0%	1	50.0%	0	0.0%	0	0.0%	0	0.0%	2	1.8%
Total	35	30.7%	47	41.2%	27	23.7%	5	4.4%	0	0.0%	114	100.0%

The chi-square test further indicates that there was a significant difference in synchronized physiological arousal episodes frequency when faced with diverse challenges (χ^2^ = 10.560, df = 2, *p* = 0.005). That is, when faced with socio-emotional challenges, the synchronized physiological arousal situations occurred the most (19, 45.24%), followed by cognitive challenges (11, 22.92%), and finally motivational challenges (2, 9.09%) (Table 5).

**TABLE 5:** Differences in synchronized physiological arousal episodes frequency under different challenges.

	Non-synchronized physiological arousal episode		Synchronized physiological arousal episode		χ^2^	*P*
	Frequency	%	Frequency	%		
CC	37	77.08%	11	22.92%	10.560	0.005
SC	23	54.76%	19	45.24%		
MC	20	90.91%	2	9.09%		

### 4.3. What are the differences in synchronized physiological arousal episodes when groups engage in different social regulation focuses (e.g., metacognitive vs. socio-emotional regulation)?

Table 6 shows the frequency and percentage of social regulation episodes with different arousal numbers. Overall, there were 444 social regulation episodes during CA, and the distribution of socio-emotional regulation (SR, 212, 47.7%) and metacognitive regulation (MR, 232, 52.3%) was relatively balanced.

**TABLE 6:** Frequency and percentage of social regulation episodes with different arousal numbers (from none to four members).

	None		1		2		3		4		Total	
	f	%	f	%	f	%	f	%	f	%	f	%
SR	57	26.9%	77	36.3%	68	32.1%	10	4.7%	0	0.0%	212	47.7%
MR	74	31.9%	100	43.1%	51	22.0%	7	3.0%	0	0.0%	232	52.3%
Total	131	29.5%	177	39.9%	119	26.8%	17	3.8%	0	0.0%	444	100.0%

The chi-square test further indicates that there was a significant difference in synchronized physiological arousal episodes frequency when engaged in different social regulation focuses (χ^2^ = 7.250, df = 2, *p* = 0.007) as the synchronized physiological arousal happened more frequently when engaged in socio-emotional regulation (78, 36.79%) rather than in metacognitive regulation (58, 25%) (Table 7).

**TABLE 7:** Differences in synchronized physiological arousal episodes frequency under different social regulations.

	Non-synchronized physiological arousal episode		Synchronized physiological arousal episode		χ^2^	*P*
	Frequency	%	Frequency	%		
SR	134	63.21%	78	36.79%	7.250	0.007
MR	174	75.00%	58	25.00%		

### 4.4. How does synchronized physiological arousal relate to the challenge being regulated?

Of 112 challenges, more than half challenges were regulated (63, 56.3%). The chi-square test further indicates that there was a significant difference in synchronized physiological arousal episodes frequency between the challenge being regulated and not being regulated (χ^2^ = 4.444, df = 2, *p* = 0.035). This is, in challenges being regulated episodes, more synchronized physiological arousal situations occurred (23, 35.48%) rather than in challenges not being regulated episodes (9, 18.37%) (Table 8).

**TABLE 8:** Differences in synchronized physiological arousal episodes frequency between the challenge being regulated and the challenge not being regulated.

	Non-synchronized physiological arousal episode		Synchronized physiological arousal episode		χ^2^	*P*
	Frequency	%	Frequency	%		
Challenge being regulated	40	64.52%	23	35.48%	4.444	0.035
Challenge not being regulated	40	81.63%	9	18.37%		

## 5. Discussion

The current research aims to utilize HR to profile the synchronized physiological arousal and further explore how the synchronized physiological arousal is related to the social regulatory process in terms of two important perspectives identified in previous research, encountering challenges and social regulation focus. Toward that end, this study developed an arousal-regulation analysis platform, which could automatically detect synchronized physiological arousal episodes in HR and align them with coding challenges and social regulation based on the timeline. After applying it in a CA activity, several findings were obtained.

First, during the CA, only one-third of the episodes were synchronized physiological arousal episodes, and the situations where four members were all in arousal states were rare. Similar findings can be found in Pijeira-Díaz et al.’s (2019) study, in which the arousal was manifested by the EDA signal. They found that only in a small part of the time (≈5–40% of the lesson), the triad members were at the same arousal levels, and the time triad members were simultaneously in high arousal was rare. Similar findings not only verify that synchronized physiological arousal is hard to achieve in collaboration (Pijeira-Díaz et al., 2018) but also prove that the methods using HR to profile arousal in this study can also be an effective approach to detecting synchronized physiological arousal.

Second, the synchronized physiological arousal occurred differently under diverse challenges. Both cognitive and socio-emotional challenges were dominant challenges encountered by students, and when faced socio-emotional challenges, synchronized physiological arousal happened more often. Some recent studies highlighted the importance of socio-emotional challenges in collaboration for the reason that, unlike cognitive challenges, socio-emotional challenges could affect the group climate (Bakhtiar et al., 2018) and, if not appropriately regulated, they may lead to a negative climate, which is harmful and unworkable for accomplishing the shared goal (Isohätälä et al., 2018). This study further signifies the importance of socio-emotional challenges by providing more objective evidence from physiological measures as collaborating members expressed more synchronized physiological responses to socio-emotional challenges (Järvelä et al., 2019a). On the contrary, synchronized physiological arousal occurred the least under motivational challenges. This result could be explained by previous findings that the unequal participation of team members is often overlooked because students are more concerned with completing tasks rather than other members’ engagement (Li et al., 2021). This finding extends previous research by further indicating that this disregard can also be reflected in students’ physiological signals as members showed the least synchronized physiological response to motivational challenges.

Third, although students exhibited balanced socio-emotional and metacognitive regulation behavior, synchronized physiological arousal more frequently co-occurred with socio-emotional regulation. This finding contradicted Dindar et al.’s (2020) investigation, which found that the overall physiological synchrony index of a team was only related to students’ self-report cognitive regulation, with no relationships found with emotional regulation. This inconsistency may be due to the different methods of data collection and processing. As suggested by previous research, utilizing physiological data to provide temporal information about collaboration in this study allowed for a more fine-grained analysis of temporal dynamics and patterns of social regulatory processes, thereby revealing more nuanced discoveries (Järvelä et al., 2019a; Dindar et al., 2022). Furthermore, combining video and physiological measures may more objectively reflect learners’ emotional and cognitive states in contrast to subjective self-reporting data (Roos et al., 2021). In addition, linking the findings of research questions 2 and 3, it can be unearthed that the shared physiological responses of collaborating members are more sensitive to the socio-emotional aspects of collaboration as the shared physiological arousal more frequently co-occurred with socio-emotional challenges and socio-emotional regulation. This result is also an improved answer to reflect the importance of socio-emotional aspects in collaboration. As revealed in previous investigations, students show an inability to regulate increasing tension (Sohr et al., 2018) and view the negative socio-emotional climate as a more difficult factor to control (Rogat and Linnenbrink-Garcia, 2011). The findings of this study not only reinforce the stance that relations matter in collaboration with physiological data (Järvelä and Rosé, 2022) but also suggests that shared physiological responses can be a signal of involvement in socio-emotional aspects of collaboration (Järvelä et al., 2019a).

The last significant finding is that the challenge being regulated episodes more frequently co-occurred with synchronized physiological arousal, which indicates a correlation between synchronized physiological responses with the challenges being regulated. Encountering challenges are a meaningful moment in collaborative learning because, if not appropriately regulated, the challenges could be detrimental to collaboration (Hadwin et al., 2018b). Although previous research has argued that the lack of regulation in responding to challenges is normally due to students’ unawareness of challenges (Järvelä et al., 2019b), there is little evidence, possibly because data on this underlying process are hard to capture. The preliminary finding of the current study can be a piece of evidence by revealing that students showed less synchronized physiological responses to the challenges not being regulated.

## 6. Conclusion and pedagogical implications

The current study developed an automatic arousal-regulation analysis platform, which contributes to the existing methods of studying the social regulatory processes by successfully utilizing HR to profile synchronized physiological arousal. After applying it in a CA activity, some important characteristics were revealed, which could provide a better understanding and facilitate superior scaffolding of the social regulatory process. For example, only one-third of episodes were synchronized physiological arousal episodes, and they were more associated with the challenges being regulated. Therefore, scripts or awareness tools are needed to help students better identify challenges and become more aware of their occurrence to respond to and regulate them promptly. In addition, the least synchronized physiological arousal under the motivation challenge should attract educators’ notice for the reason that this disregard for equal participation could lead to poor group performance in the long term (Li et al., 2021). Instructional intervention, such as directly emphasizing the value of equal participation, should be implemented to guide students to pay more attention to group members’ engagement. Finally, synchronized physiological arousal is more sensitive to socio-emotional challenges as well as socio-emotional regulation. This finding not only reinforces the importance of socio-emotional aspects in collaboration but also calls for support to help students cope with socio-emotional challenges and facilitate socio-emotional regulation, thereby maintaining a favorable socio-emotional atmosphere.

## 7. Limitations and future work

The main limitation of this study is the sample size, uneven gender ratio, and the unitary learning context. As physiological measures can be affected by contextual changes (Järvelä et al., 2019a), the preliminary findings, as well as the function of the arousal-regulation analysis platform, need to be further validated in a larger, more gender-balanced sample and applied in more contexts. Furthermore, to answer the research question, the present study used 30 s as the analysis unit of HR to align with the videos and explore synchronized physiological arousal under different events. Future research can first identify the synchronized physiological arousal episodes without considering the video and then analyze the behavioral characteristics under these episodes in reverse. Finally, while arousal has been shown to be positively related to an individual learners’ performance (Pijeira-Díaz et al., 2018), and there is also a consensus on the value of synchronized physiological arousal in collaboration (Dindar et al., 2022), empirical research on the relationship between synchronized physiological arousal and group performance is still scarce.

## Data availability statement

The raw data supporting the conclusions of this article will be made available by the authors, without undue reservation.

## Ethics statement

The research involved human participants and followed the procedures of the Education Faculty’s Ethics Committee. Informed consent was obtained for all participants.

## Author contributions

XL led the research project, analyzed the data, and wrote the manuscript. WH conducted the data collection and analysis. YL supervised the research and revised the manuscript. ZM supported the instructional process. All authors read and approved the final manuscript.

## Footnotes

1
https://www.processon.com
